# A two-stage deep learning prediction system for colon cancer microsatellite instability status using CT images

**DOI:** 10.3389/fonc.2025.1699430

**Published:** 2026-01-12

**Authors:** Songlin Cui, Xin Xiong, Xudong Yang, Jianfeng He, Tao Shen

**Affiliations:** 1 Faculty of Information Engineering and Automation, Kunming University of Science and Technology, Kunming, China; 2 Department of Colorectal Surgery, The Third Affiliated Hospital of Kunming Medical University, Kunming, China

**Keywords:** microsatellite instability, colon cancer, deep learning, segment anything, LORA, contrastive learning

## Abstract

**Background:**

This study seeks to build a two-stage deep learning approach for identifying the microsatellite instability (MSI) status of colon cancer based on computed tomography (CT) scans without the requirement for manual segmentation.

**Methods:**

This study included 108 enhanced CT scans of colon cancer, including 68 cases of ascending colon, 14 cases of transverse colon, 18 cases of descending colon, and 8 cases of sigmoid colon; there were 56 cases of MSI-H and 52 cases of microsatellite stability (MSS). In the first stage, the segmentation model MSI-SAM was trained to accurately segment the lesion locations in the CT scans. In the second stage, the mask acquired from the MSI-SAM segmentation was multiplied by the original CT image (CT_Origin) bitwise, and the result was merged with the mask obtained from the MSI-SAM segmentation (Segment) to obtain CT_ROI. Both CT_ROI and CT_Origin were then diagnosed using the colon cancer MSI status diagnosis model.

**Results:**

The performance of the suggested CT segmentation model MSI-SAM in the ascending colon, transverse colon, descending colon, and sigmoid colon areas (DSC: IoU) was (0.886:0.798), (0.878:0.783), (0.923:0.857), and (0.854:0.747), respectively. The AUC of the MSI status diagnostic model for patients with colon cancer was 0.935 (95% CI 0.892–0.947), the ACC was 0.913, the sensitivity was 1.000, and the specificity was 0.846.

**Conclusions:**

The segmentation masks created by the trained deep learning segmentation model achieved a level comparable to that of expert radiologists, and the deep learning diagnostic model played an essential role in supporting doctors in diagnosis.

## 1 Introduction

Studies have revealed that patients with colon cancer with high microsatellite instability (MSI-H) do not react to 5-fluorouracil chemotherapy but are susceptible to immunotherapy and have a good prognosis in the early stages (1, 2). Therefore, precisely detecting the MSI status of patients with colon cancer is crucial for clinical therapy and prognosis. Routine testing for MSI includes immunohistochemistry (IHC), polymerase chain reaction (PCR), and next-generation sequencing (NGS) (3). However, relatively few medical institutions are equipped with these tests, the NGS method is expensive and technically demanding, and not suitable for stand-alone MSI testing (4), and IHC results may be interfered with by benign germline polymorphisms, leading to false-negative and false-positive results. Not only that, all of these tests are invasive and need surgery or biopsy to obtain tissue specimens. Therefore, there is an urgent need for a noninvasive preoperative screening approach to predict the MSI status of patients with colon cancer and to guide accurate and customized treatment.

Computed tomography (CT) is a noninvasive imaging modality that is widely used in the clinical practice of colon cancer (5). Pernicka et al. (6) created three machine learning prediction models comprising clinical features, imaging histology features, and a mix of the two by evaluating preoperative CT images of 198 patients with stage II–III colon cancer; Gao et al. (7) analyzed enhanced CT images of 108 patients with colon liver metastasis and selected 7 imaging histological features that can effectively distinguish MSI-H from microsatellite stability (MSS), and used a random forest model for classification, and Ma et al. and Pei et al. (8, 9) used a similar method for MSI prediction. The preceding approach for MSI status detection based on colon cancer CT by imaging histology is halted; i.e., after extracting the features through radiomics (10), we need to manually screen the features to form the model for training. Radiomics relies on hand-designed features, like grayscale covariance matrix, stroke length matrix, and other features (10), and the selection and construction of these features are heavily dependent on the researcher’s experience and past knowledge.

Recently, the advancement of deep learning (DL) has led to major advances in computer-aided diagnosis (CAD) in the field of medical imaging (11), with extensive applications across diverse tasks including few-shot learning, histopathology analysis, and biometric recognition (12–15). Compared with traditional medical imaging diagnosis, the feature extraction technique in DL can tap into the latent information that cannot be detected by the naked eye. Therefore, CT-based DL approaches are of tremendous aid in the prediction of MSI status in patients with colon cancer and the formulation of customized treatment programs for patients after physicians. To this purpose, we present a two-stage DL colon cancer MSI diagnostic technique based on segmentation followed by diagnosis.

This colon cancer MSI status diagnostic approach avoids the time-consuming and labor-intensive procedure of human segmentation of CT. In the final experiment, we use the mask Segment obtained from MSI-SAM segmentation to outperform the CT-based diagnostic methods in colon cancer MSI status diagnosis, which focuses on the local region of the ROI and takes into account the global CT information to better help the colon cancer MSI status diagnostic model to make a decision, and also proves that Segment has a comparable performance with the mask sketched by the doctor in colon cancer MSI status diagnosis. It also indicates that Segment and doctor-drafted masks have equivalent performance in colon cancer MSI status diagnosis.

## 2 Materials and methods

### 2.1 Data description and dataset division

All CT data in this study were acquired using a SOMATOM Definition AS+64-slice 128-layer spiral CT scanner manufactured by Siemens Healthineers. Scan parameters were set as follows: tube voltage: 120 kV; tube current: CareDose 4D intelligent dose modulation enabled; spiral pitch: 0.6; and image reconstruction slice thickness: 2 mm. The scanning range covered the entire abdomen, specifically from 2 cm above the diaphragm to the lower margin of the symphysis pubis. Contrast agent administration employed a dual-phase bolus injection protocol using a dual-chamber high-pressure syringe. The contrast agent used was iopamidol (concentration, 300 mgI/mL), administered at a dose of 1.2 mL/kg with an infusion rate of 3.0–3.5 mL/s. Following the contrast agent bolus, 30 mL of normal saline was infused at the same rate to ensure adequate distribution. The scanning sequence comprised three phases: the arterial phase initiated 30–35 s after contrast injection, the parenchymal phase began 80–85 s post-injection, and the excretory phase commenced 15–30 min post-injection. All acquired images were retrieved and exported from the Picture Archiving and Communication System (PACS).

There were a total of 108 CT images of colon cancer, comprising 68 cases of ascending colon, 14 cases of transverse colon, 18 cases of descending colon, and 8 cases of sigmoid colon; there were 56 cases of patients with colon cancer with MSI-H status, and 52 cases of patients with colon cancer with MSS status.

During the CT segmentation phase for colon cancer, CT data from different regions of colon cancer were divided into an 8:2 ratio to form the training and testing datasets. In the MSI status diagnosis phase, CT data from colon cancers with different MSI statuses were similarly divided into an 8:2 ratio to constitute the training and testing datasets. To characterize the study population and ensure the reliability of the experimental results, we summarized the demographic and clinical baseline characteristics of the 108 enrolled patients in **Table 1**.

**Table 1:** The demographic and clinical baseline characteristics of the 108-patient cohort, including age, gender, tumor location, and MSI status, showed a balanced distribution between MSI-H and MSS.

Characteristic	Total cohort (*n*=108)	MSI-H (*n*=56)	MSS (*n*=52)
Age, years	56.67 (37–77)	51.07 (37–71)	62.69 (40–77)
Sex, *n* (%)			
Male	53 (49.1)	24 (42.9)	21 (40.4)
Female	55 (50.9)	32 (57.1)	31 (59.6)
Tumor location, *n* (%)			
Ascending colon	68 (63.0)	37 (66.1)	31 (59.6)
Transverse colon	14 (13.0)	5 (8.9)	9 (17.3)
Descending colon	18 (16.6)	10 (17.9)	8 (15.4)
Sigmoid colon	8 (7.4)	4 (7.1)	4 (7.7)
MSI status, *n* (%)			
MSI-H	56 (51.9)	—	—
MSS	52(48.1)	—	—

### 2.2 LoRA on the Image Encoder3D of MSI-SAM

Identifying MSI-related regions and extracting valuable information from complex colon cancer CT images can greatly avoid the colon cancer MSI status diagnosis model from not capturing the valid information in the CT images; thus, in the segmentation stage of colon cancer CT images, the MSI-SAM model is trained to segment the ROI of the colon cancer CT images.

**Figure 1:**
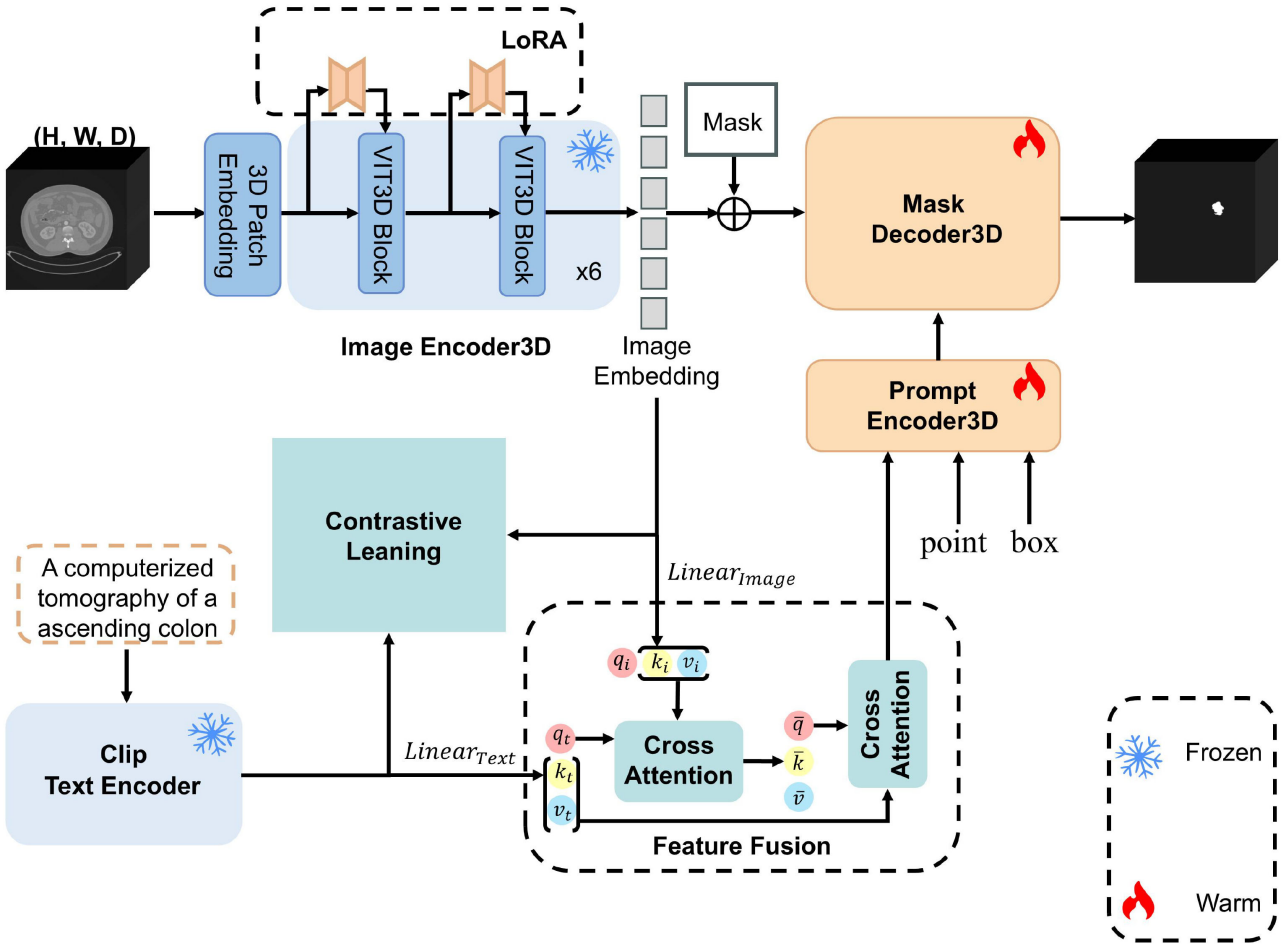
Overview of the MSI-SAM CT segmentation model for colon cancer.

Our colon cancer CT segmentation model MSI-SAM is shown in **Figure 1**. The structure of MSI-SAM is inherited from that of SAM-Med3D; the dimension of image processing is three-dimensional, which solves the disadvantage that SAM cannot deal with the spatial information of medical images, and likewise the parameter of the whole model focuses on the Image Encoder3D part. The colon cancer CT that needs to be segmented is outputted as an image feature representation after the Patch Embedding operation through multiple Vision Transformers (VITs) (16), before which the pre-training weights need to be loaded and frozen, and we describe the pre-training weights in the Image Encoder3D part as follows. The LoRA fine-tuning approach is achieved by adding a bypass to the frozen Transformer structure, which consists of two linear layers, $B\in {\mathbb{R}}^{C_{in}\;x\;r\;}$ and $A\in {\mathbb{R}}^{r\;x\;C_{out}}$, where r≪min{Cin, Cout}, and the updated weights are Equation 1:

(1) $$\bar{W}=W+\Delta W=W+\;BA$$

Consider that the input sequence of Image Encoder3D is $x\in {\mathbb{R}}^{\text{HxWxD}}$, and the output following Image Encoder3D that has been fine-tuned by LoRA low rank is Equation 2:

(2) $$Z_{I}=\bar{W}x=(W+\Delta W)x=(W+BA)x$$

Thanks to the prompt learning of the SAM model and the comparative learning (CL) of the Clip (17) model, we execute a semantic prompt for distinct colon cancer sites. Clip and SAM-Med3D are the same pre-trained model, which collects images a huge number of image–text pairs for pre-training, and builds a connection between the images and the text. Following the training approach of Clip, we performed an alignment operation between the text feature representation extracted by Text Encoder in Clip and the image feature representation $Z_{i}$ extracted by Image Encoder3D in MSI-SAM using the Info NEC (18) loss function. Contrast learning allows the model to learn to distinguish between similar and dissimilar data samples (19), stating that each CT corresponds to a positive sample location prompt text representation as $Z_{T+}$ and a negative sample as $Z_{T-}$. Using the cosine similarity, sim measures the similarity between the two modal representations, and the more similar the current CT is to the positive sample textual representation against the more unlike it is to the negative sample, the lesser the loss will be. Info NEC adds a temperature coefficient T to the NEC loss function. Info NEC adds a temperature coefficient *E* to the NEC loss function, which boosts the model’s capacity to discriminate between negative samples, allowing the model to focus on negative cases that are harder to identify from positive ones. Info NEC loss function is Equation 3:

(3) $$L_{\text{Info NEC}}=-\frac{1}{N}\sum_{i=1}^{N}\text{log}\frac{\text{exp}(\text{sim}(Z_{I},Z_{T+}))/T}{\text{exp}(\text{sim}(Z_{I},Z_{T+}))/T+\sum_{N\in Z_{t-}}\text{exp}(\text{sim}(Z_{I},N))/T\;}$$

### 2.3 Feature fusion between CT images and positional text

After CL alignment, the positional text feature representation output by Text Encoder is similar to that of the corresponding image in terms of data distribution, but the aligned positional text feature representation directly input into the prompt encoder of MSI-SAM for positional prompt not only will not help the segmentation effect but also will cause trouble to the model obtained by LoRA fine-tuning, resulting in the segmentation effect of model degradation (20). To make the aligned textual representations better understand the semantic information in the images and provide more accurate location prompt for different colon cancer CT sites, we use the feature fusion (FF) module implemented through the cross-attention mechanism to fuse the CT image feature representations with the corresponding location textual feature representations to better prompt different colon cancer sites. In the FF module, two separate modal feature representations have their corresponding qkv for producing cross-attention, and the related formulas are as follows (Equation 4):

(4) $$I_{q,k,v},T_{q,k,v}=\mathrm{Linea}{\text{r}}_{q,k,v}^{I|T}(Z_{I}Z_{T})$$

FF consists of two cross-attention modules, ${\text{CA}}_{1}$ and ${\text{CA}}_{2}$. The ${\text{CA}}_{1}$ inputs are $T_{q}$, $I_{k}$, and $I_{v}$. The ${\text{CA}}_{1}$ module formula is as follows (Equation 5):

(5) $${\text{CA}}_{1}(T_{q},I_{k},I_{v})=\text{SoftMax}(\frac{T_{q}I_{k}^{T}}{\sqrt{d_{I_{k}}}})I_{v\;}$$

When the first cross-attention module ${\text{CA}}_{1}$ of the FF, we may acquire $\bar{q}$, $\bar{k}$, and $\bar{v}$, which contain the information of two modal feature representations, and then when the second cross-attention module ${\text{CA}}_{2}$ is fully fused, the formula is as follows (Equation 6):

(6) $${\text{CA}}_{2}(\bar{q},T_{k},T_{v})=\text{softmax}(\frac{\bar{q}T_{k}^{T}}{\sqrt{d_{T_{k}}}})T_{v}$$

The feature representations of the two modalities are fully fused through the FF module and input into the Prompt Encoder 3D module of MSI-SAM to provide corresponding text position prompts for colon cancer CT.

### 2.4 Employ KAN to replace MLP in MSI classification

**Figure 2:**
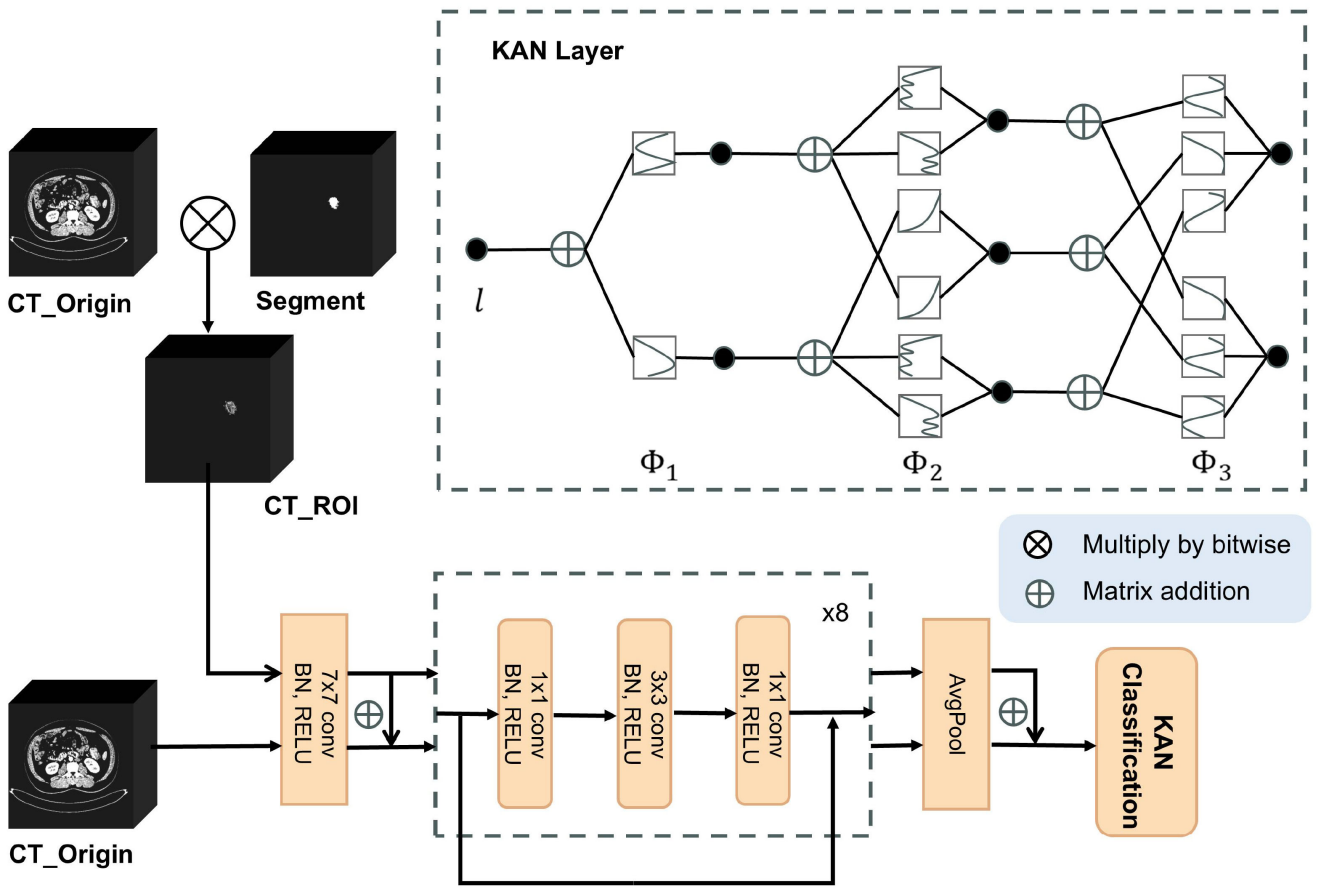
Overview of diagnostic methods for MSI status in patients with colon cancer.

Our colon cancer MSI status diagnosis model is displayed in **Figure 2**. The feature extraction part of the whole network architecture is inherited from ResNet18, and its added residual connections strengthen the connection between different layers of the network, avoiding the gradient disappearance or gradient explosion during training, and solving the degradation problem of the deep network during training. The information related to the MSI status is mainly contained in the ROI in the CT of colon cancer, and we use the mask Segment obtained by MSI-SAM segmentation and the corresponding CT_Origin to multiply by bit to get a copy of CT_ROI containing only the CT information of the ROI, and CT_Origin containing all the information provides a wider perspective for observing the MSI; thus, CT_ROI and CT_Origin are input into the improved ResNet18 colon cancer MSI status diagnosis model in parallel, and matrix summation is performed after the convolution operation with a convolution kernel size of 7 and the global average pooling operation afterward to achieve FF under different CT perspectives during the feature extraction process, respectively.

Meanwhile, to tackle the difficulties of poor parameter utilization efficiency and poor interpretability that normally exist in MLP networks, the final MLP classification output layer of the MSI status diagnostic network is substituted by a KAN network. As shown in **Figure 2**, the design principle of KAN originates from the Kolmogorov–Arnold theorem (21), and KAN differs from MLP in that, although it also possesses a fully connected structure, there is no linear weight matrix; instead, each weight parameter is replaced by a learnable one-dimensional function parameterized by a spline. In the nodes of a KAN, the incoming signals are merely subjected to a basic summation operation without any nonlinear transformations. KAN is typically able to realize smaller computational graphs than MLP (22). After the convolution operation of the ResNet18 network and the fusion of the two CT feature summations, the recovered image features are marked as *I*. Finally, the KAN is utilized to make the final diagnosis of the MSI status of the colon cancer patient. In a KAN network, denoting the whole design of the *i*th layer, a KAN with *I* layers can be stated as Equation 7:

(7) $$\text{KAN}(I)=({\text{Φ}}_{k}⊗{\text{Φ}}_{k-1}⊗{\text{Φ}}_{k-2}\cdots \cdots {\text{Φ}}_{1})I$$

Therefore, to better align with the feature extraction component of ResNet18, we set the number of layers *I* in KAN to match the number of layers in the fully connected component of ResNet18.

## 3 Results

### 3.1 Model evaluation

In the colon cancer CT segmentation challenge, we employed DSC and IoU to accomplish the evaluation, with the experimental results at this stage obtained through fivefold cross-validation. Similarly, to comprehensively evaluate the performance of the MSI status diagnostic model for colon cancer, we used AUC, ACC, sensitivity, and specificity as evaluation metrics, and the experimental results of this stage were also derived via fivefold cross-validation. The DSC and IoU formulas are as follows (Equations 8, 9), where *A* is the set of predicted results and *B* is the set of true labels:

(8) $$\text{DSC}=\frac{2\times |A\cap B|}{\left|A|+|B\right|}$$

(9) $$\text{IoU}=\frac{\left|A\cap B\right|}{\left|A\cup B\right|}$$

### 3.2 Segmentation results

#### 3.2.1 Comparison with other SAM pre-trained methods

To validate the performance of MSI-SAM on our dataset, we introduced two categories of comparative models to ensure a comprehensive evaluation: (1) non-SAM-based clinical DL baselines widely used in 3D medical image segmentation, including 3DUNet (23) and 3DTransUNet (24); (2) SAM-based large medical models capable of handling 3D data, including SAM-Med3D (25), Promise (26), FastSAM3D (27), and 3DSAM (28). For the SAM-based models, we loaded their corresponding pre-trained weights, while 3DUNet and 3DTransUNet were trained from scratch using the same training protocol. All comparative networks and the proposed MSI-SAM were subjected to the identical preprocessing pipeline and evaluated on the same dataset partition (8:2 training–test split). The comparison results are shown in **Table 2**: MSI-SAM achieves a DSC of 0.886 and an IoU of 0.798 on the ascending colon, with DSC–IoU values of 0.878–0.783 (transverse colon), 0.923–0.857 (descending colon), and 0.854–0.747 (sigmoid colon), outperforming both non-SAM-based baselines and SAM-based models across all colon sites.

**Table 2:** Quantitative comparison of MSI-SAM with other SAM pretraining methods for segmenting 3D medical images at different sites on the CT dataset of colon cancer.

Methods	Ascending		Transverse		Descending		Sigmoid	
	DSC	IoU	DSC	IoU	DSC	IoU	DSC	IoU
3DUNet (23)	0.844	0.750	0.852	0.763	0.866	0.782	0.798	0.723
3DTransUNet (24)	0.865	0.765	0.863	0.777	0.878	0.802	0.835	0.745
SAM-Med3D (25)	0.746	0.600	0.728	0.572	0.749	0.604	0.587	0.416
Promise (26)	0.445	0.315	0.237	0.153	0.375	0.262	0.649	0.481
FastSAM3D (27)	0.673	0.511	0.617	0.450	0.715	0.558	0.621	0.455
3DSAM (28)	0.541	0.397	0.353	0.258	0.686	0.532	0.465	0.307
MSI-SAM	**0.886**	**0.798**	**0.878**	**0.783**	**0.923**	**0.857**	**0.854**	**0.747**

The bold values in this table represent the best performance achieved across all methods for the corresponding evaluation metrics (DSC, IoU) in the segmentation task of each anatomical site.

**Figure 3:**
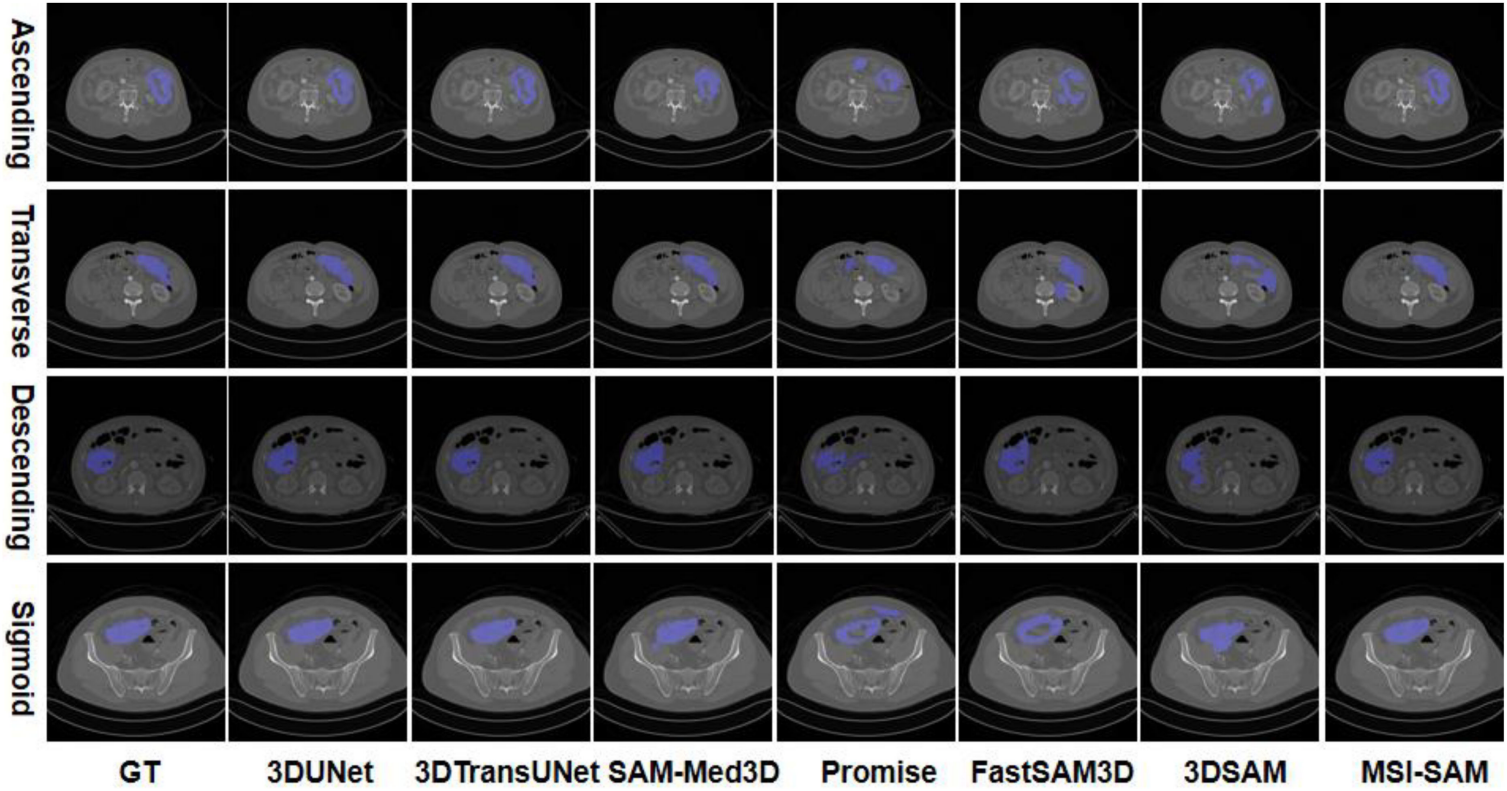
Qualitative visualization of the proposed method, MSI-SAM with benchmark methods on CT of four colon cancer sites: ascending colon, transverse colon, descending colon, and sigmoid colon. The benchmark approaches include 3DUNet (23), 3DTransUNet (24), SAM-Med3D (25), Promise (26), FastSAM3D (27), and 3DSAM (28).

To intuitively illustrate the segmentation performance, we present the segmentation results of different algorithms across four colon cancer sites (ascending colon, transverse colon, descending colon, and sigmoid colon) in **Figure 3**. In the visualization, the colon cancer lesion is marked in blue as the segmented foreground, while varying grayscale values represent the background. As shown, compared to both non-SAM-based clinical baselines (3DUNet and 3DTransUNet) and SAM-based 3D medical image segmentation models (SAM-Med3D, Promise, FastSAM3D, and 3DSAM) that have been pre-trained on large medical datasets, our MSI-SAM network—fine-tuned on our specified dataset—achieves more complete lesion region segmentation and better edge integrity. For instance, 3DUNet and 3DTransUNet exhibit partial under-segmentation or irregular boundaries, while SAM-Med3D, Promise, FastSAM3D, and 3DSAM either miss lesion details or show fragmented segmentation. In contrast, MSI-SAM consistently aligns with the ground truth (GT) in contour completeness and edge accuracy across all four colon sites.

#### 3.2.2 CT segmentation ablation experiment

To prove the improvement effect of MSI-SAM more fully, ablation experiments were done on LoRA fine-tuning, text-image alignment (CL), and text-image fusion modules (FF), respectively, and the experimental results are provided in **Table 3**. **Table 3** proves the influence of each module chosen in this research on boosting the capability of MSI-SAM in CT segmentation of colon cancer. The MSI-SAM model obtained by fine-tuning SAM-Med3D with the LoRA strategy demonstrated significantly improved performance on the colon cancer CT dataset. Testing different values for *r* revealed that fine-tuning yielded optimal results when *r* was set to 8. Because of the larger volume of CT data in the ascending colon, MSI-SAM outperformed the other three regions in both DSC and IoU evaluation metrics.

**Table 3:** Quantitative results of MSI-SAM ablation analysis with different components.

Methods	Ascending		Transverse		Descending		Sigmoid	
	DSC	IoU	DSC	IoU	DSC	IoU	DSC	IoU
LoRA *r*=2	0.846	0.788	0.763	0.663	0.855	0.788	0.792	0.688
LoRA *r*=4	0.872	0.803	0.794	0.688	0.893	0.819	0.811	0.696
LoRA *r*=8	**0.896**	**0.812**	0.817	0.695	0.904	0.826	0.828	0.708
LoRA *r*=16	0.884	0.809	0.804	0.692	0.895	0.823	0.821	0.698
LoRA (*r*=8)+CL	0.762	0.544	0.680	0.517	0.778	0.605	0.743	0.533
LoRA (*r*=8)+CL+FF	0.886	0.798	**0.878**	**0.783**	**0.923**	**0.857**	**0.854**	**0.747**

The bold values in this table represent the best performance achieved across all methods for the corresponding evaluation metrics (DSC, IoU) in the segmentation task of each anatomical site.

Simply inputting text aligned with corresponding CT scans into the MSI-SAM Prompt Encoder3D for text-based positional guidance actually degrades the performance of models fine-tuned with LoRA. Considering that text features and CT image features were not fully understood, after integrating both types of features through the FF module, the model achieved optimal segmentation performance on both DSC and IoU metrics.

### 3.3 Diagnostic results

#### 3.3.1 Diagnosis of MSI status in colon cancer and ablation experiments

In the process of colon cancer MSI status diagnosis, we conducted ablation experiments to explore how different strategies affect the performance of the ResNet18-based diagnostic model, with results presented in **Table 4**. For the baseline native ResNet18 model that only takes single-input CT_Origin (global abdominal CT information), its diagnostic performance is limited—achieving an AUC of 0.810, an ACC of 0.783, a sensitivity of 0.800, and a specificity of 0.769. This limitation arises because 3D CT data contain extensive non-MS-related background information, making it difficult for the single-input model to focus on lesion regions critical to MSI status judgment.

**Table 4:** Ablation trials utilizing three techniques, Segment, KAN, and Mask, on the impact of diagnostic results of MSI status in colon cancer.

Strategies	AUC (95% CI)	ACC (95% CI)	Sensitivity (95% CI)	Specificity (95% CI)
CT_Origin	0.810 (95% CI 0.732–0.838)	0.783 (95% CI 0.722–0.806)	0.800 (95% CI 0.779–0.821)	0.769 (95% CI 0.755–0.813)
+Segment	0.890 (95% CI 0.640–0.932)	0.870 (95% CI 0.825–0.901)	0.900 (95% CI 0.862–0.934)	0.846 (95% CI 0.798–0.911)
+Segment, KAN	0.935 (95% CI 0.892–0.947)	0.913 (95% CI 0.870–0.957)	1.000 (95% CI 0.932–1.000)	0.846 (95% CI 0.821–0.894)
+Mask, KAN	0.943 (95% CI 0.922–0.986)	0.913 (95% CI 0.870–0.957)	0.900 (95% CI 0.885–0.964)	0.923 (95% CI 0.902–0.966)

To address this, we introduced the segmentation mask (Segment) output by the MSI-SAM model (from the first segmentation stage) to construct CT_ROI (lesion-local information) via bitwise multiplication with CT_Origin. At this point, the model transitions to a dual-input framework that integrates CT_Origin (global anatomical context) and CT_ROI (targeted lesion details). With this dual-input design, the model’s extracted features simultaneously cover full CT information and MSI-relevant lesion regions, leading to notable performance improvements.

Furthermore, replacing the final MLP layer of ResNet18 with KAN in this dual-input framework yielded the optimal diagnostic results: an AUC of 0.935, an ACC of 0.913, a sensitivity of 1.000, and a specificity of 0.846. This confirms that the combination of dual-input FF (CT_Origin+CT_ROI) and KAN’s superior feature mapping capability is key to enhancing the model’s MSI status diagnostic accuracy.

To clarify the statistical significance of performance differences between models in the second-stage MSI diagnosis phase, we present **Table 5**, which quantifies comparisons between our proposed dual-input KAN model [Dual-input(Segment)+KAN] and baseline models using significance testing.

**Table 5:** Quantitative comparison and significance testing of MSI diagnostic models in the second stage.

Comparison (proposed model vs. baseline)	Metric	Proposed model	Baseline model	*p*-value	Significance
Dual-input (Segment)+KAN vs. Single CT_Origin+MLP	AUC	0.935	0.810	0.002	*p*<0.01
	ACC	0.913	0.783	0.038	*p*<0.05
Dual-input (Segment)+KAN vs. Dual-input+MLP (Segment)	AUC	0.935	0.890	0.018	*p*<0.05
	ACC	0.913	0.870	0.049	*p*<0.05
Dual-input (Segment)+KAN vs. Single CT_Origin+MLP (Mask)	AUC	0.935	0.943	0.620	–
	ACC	0.913	0.913	1.000	–

The table evaluates three key comparison scenarios, reporting metrics (AUC and ACC), *p*-values, and significance levels (with *p*<0.05 and *p*<0.01 denoting statistical significance). The test methods are DeLong test for AUC and McNemar’s test for ACC, with Bonferroni correction applied for multiple comparisons. Compared to Single CT_Origin+MLP, our proposed model shows highly significant improvements in both AUC (12.5% increase, *p*<0.01) and ACC (13% increase, *p*<0.05), validating the value of dual-input fusion (CT_Origin+CT_ROI). When replacing MLP with KAN in the dual-input framework [Dual-input+MLP(Segment)], our model still achieves significant gains in AUC (4.5% increase, *p*<0.05) and ACC (4.3% increase, *p*<0.05), highlighting KAN’s superiority in parameter efficiency and feature alignment.

The key is that our dual input KAN model showed no statistical difference between using different masks drafted by radiologists and MSI-SAM automatic segmentation, which confirms that automatic segmentation is equivalent to manual annotation in clinical practice, thus meeting the needs of low-labor, noninvasive MSI diagnosis.

**Figure 4:**
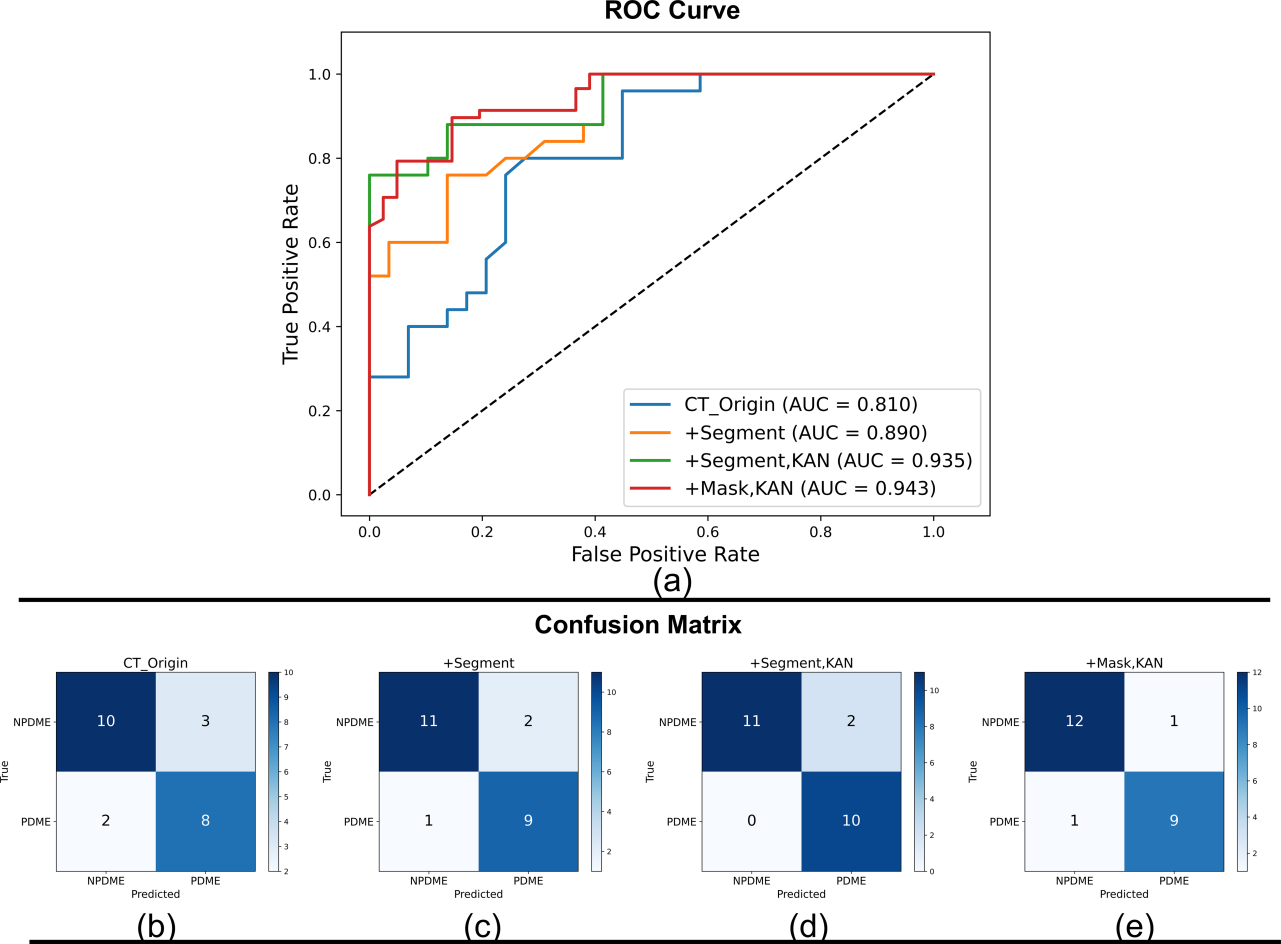
Performance of the MSI status diagnostic model for colon cancer under different conditions. **(a)** ROC curves of the MSI diagnostic model under different conditions. **(b)** Confusion matrix based only under CT_Origin. **(c)** Confusion matrix after adding Segment segmented by MSI-SAM. **(d)** Confusion matrix after adding Segment and replacing MLP with KAN. **(e)** Confusion matrix after adding Mask and replacing MLP with KAN.

To verify that the mask Segment of MSI-SAM segmentation has comparable performance with the mask Mask outlined by the imaging physician in the diagnosis of colon cancer MSI status, at this time, CT_ROI is derived from the multiplication of CT_Origin and Mask by bit, and the diagnostic model uses Mask based on the two evaluation indexes of AUC and sensitivity to reach 0.943 and 1.000; specificity is slightly worse and comparable to ACC, but overall, the mask of MSI-SAM segmentation has a comparable performance to the mask sketched by the imaging physician in the diagnosis of colon cancer MSI status. We also demonstrate the outcomes of the ablation experiments for the colon cancer MSI status diagnosis job under different techniques from another perspective. We drew the ROC curves employing different strategies in **Figure 4**, and the results corresponding to **Table 3** can be seen in the figure.

**Figure 5:**
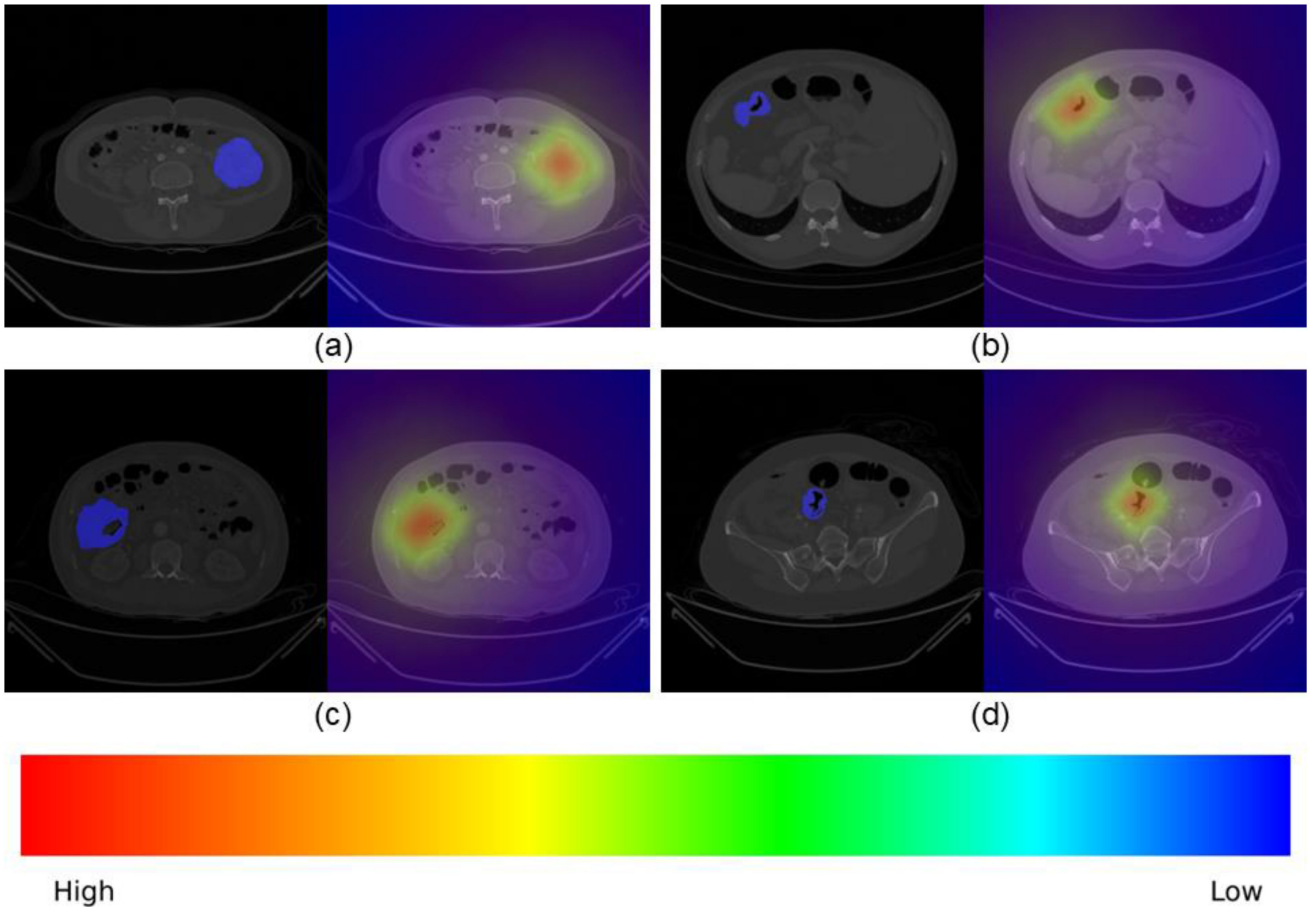
For each site, the left panel displays the original CT image covering the lesion mask (blue), marking the areas of interest, while the right panel displays the Grad CAM heatmap, where the color gradient from red (high attention) to blue (low attention) represents the model’s priority for MSI-related features. **(a)** Ascending colon, **(b)** transverse colon, **(c)** descending colon, and **(d)** sigmoid colon.

In order to meet the interpretability requirements of the model, **Figure 5** shows the Grad CAM attention heatmap visualization results of the proposed MSI diagnostic model on representative colon cancer CT images, where the red highlighted areas represent the decision key regions that the model focuses on. The four sets of images show the original abdominal CT images and corresponding Grad CAM heatmaps of MSI-H status patients in different colon cancer sites, clearly marking the boundaries of colon lesions. It can be observed that the model always focuses attention on the tumor lesion area and its adjacent intestinal wall, rather than irrelevant background tissues such as fat, muscle, or normal intestinal segments—this attention distribution is highly consistent with the clinical attention of radiologists to lesion features. This visualization not only breaks the “black box” limitations of DL, but also proves that the decision-making basis of the model is consistent with clinical diagnostic logic, laying the foundation for clinical trust and application.

## 4 Discussion

In this study, we created a colon cancer MSI status diagnostic approach based on two-stage DL, i.e., segmentation followed by diagnosis, which provides a unique solution for the clinical noninvasive diagnosis of MSI.

Inspired by the field of Natural Language Processing (NLP), SAM-Med3D (25) is proposed in the field of image segmentation and pre-trained on a fully processed large-scale 3D medical dataset. The pre-trained base model usually performs poorly in the defined application scenarios, as shown in **Table 2**, and the SAM-Med3D pre-trained model performed moderately on the untrained unfamiliar dataset and not enough to be applied to the next step of diagnosis of MSI status of colon cancer. LoRA (29) is a common and effective Parameter Efficient Fine-Tuning (PEFT) (30) method, which requires much less updating than the whole model parameters through a low-rank decomposition strategy, which greatly reduces the consumption of computational resources and decreases the computational equipment requirements.

Thus, in the CT segmentation stage of colon cancer, MSI-SAM achieved the best results in each colon cancer site after adaptation on a specific dataset, which fully demonstrates the importance of fine-tuning and customization of pre trained models in specific tasks to improve the segmentation performance of MSI-SAM models. Meanwhile, we conducted ablation experiments on the rank *r* of the LoRA fine-tuning during the segmentation phase of colon cancer CT scans. We observed that the model’s segmentation capability progressively improved as *r* increased from 2 to 8. However, when *r* reached 16, the model exhibited varying degrees of performance degradation across different colon cancer regions. Therefore, we retained *r* at 8 and proceeded to ablation experiments on other modules in the subsequent phase.

Directly inputting text position prompts into the Prompt Encoder 3D module of MSI-SAM after aligning the CL module can actually lead to a decrease in model performance. This is because although the two aligned feature representations have similar distributions in vector space, they do not fully understand each other. Therefore, after aligning the two feature representations in the CL module, they are fused and fully understood through the FF module before being input into the Prompt Encoder 3D module for text position prompts. It can be seen that the performance of the model at this point has been substantially enhanced in the transverse colon, descending colon, and sigmoid colon by the whole body of MSI-SAM at the expense of the performance of some areas of the ascending colon.

To comprehensively validate our segmentation model MSI-SAM, we included two non-SAM-based clinical baselines 3DUNet and 3DTransUNet, widely used in 3D medical image segmentation, alongside SAM-based models for comparison. As shown in **Table 2**, 3DUNet (DSC 0.798–0.866, IoU 0.723–0.782) and 3DTransUNet (DSC 0.835–0.878, IoU 0.745–0.802) outperform most SAM-based models (e.g., SAM-Med3D DSC 0.587–0.749, Promise DSC 0.237–0.649) due to their tailored 3D medical segmentation architectures. However, our MSI-SAM (DSC 0.854–0.923, IoU 0.747–0.857) still surpasses both baselines by integrating LoRA fine-tuning (*r*=8) and cross-modal FF, confirming that task-specific optimization enhances 3D segmentation adaptability for colon cancer CT.

The structural differences among comparative models further explain performance gaps: SAM-Med3D and FastSAM3D reconfigure SAM’s full architecture for 3D data, while Promise and 3DSAM only add adapters (requiring 3D data splitting/assembling), leading to suboptimal feature extraction (**Table 2**). This aligns with 3DUNet/3DTransUNet’s advantage in 3D spatial information capture, yet MSI-SAM’s superiority highlights the value of combining architecture-level 3D adaptation with LoRA and FF.

In the diagnosis stage, our ResNet18-based model replaces MLP with KAN and inputs CT_Origin and CT_ROI in parallel. **Table 5** supplements *p*-values and significance testing to verify model differences: compared to single CT_Origin+MLP, our model shows highly significant improvements in AUC (0.935 vs. 0.810, *p*=0.002) and ACC (0.913 vs. 0.783, *p*=0.038); compared to dual-input+MLP, gains in AUC (*p*=0.018) and ACC (*p*=0.049) remain significant. Additionally, **Figure 5**’s Grad-CAM attention maps confirm that the model focuses on tumor lesions, not irrelevant background, aligning with clinical diagnostic logic-breaking DL’s “black box” while validating that its decision-making basis is clinically interpretable.

The two-stage MSI diagnostic system is well-suited for integration into radiology workflows: it directly accepts standard format CT data from clinical PACS systems and uses MSI-SAM to automate lesion segmentation and the ResNet18+KAN model to process CT_Origin/CT_ROI for diagnosis without manual feature extraction—freeing radiologists to focus on high-value tasks like edge-case review. Notably, newly added clinical data can be used to continuously fine-tune MSI-SAM and retrain the diagnostic model, enabling iterative performance improvement aligned with long-term workflow use. Before deployment, key steps are required: conducting a reader study with three to five abdominal radiologists to confirm that the system enhances clinical judgment, and establishing quarterly post-deployment performance monitoring to maintain reliability.

Overall, this two-stage DL method is highly effective, fast, and reliable in diagnosing the MSI status of colon cancer, and the whole process greatly avoids human intervention such as manual segmentation, manual extraction of features, and screening of colon cancer CT, which provides strong support for clinicians to develop personalized and precise treatment plans. However, there are some drawbacks in this work, such as the relatively small size of the dataset, which may impair the generalization capacity of the model, and the diagnostic effect of different colon cancer locations was not studied in the second stage of the diagnostic approach. Future studies can further expand the sample size, study the application of the model in different clinical circumstances, and continually enhance the performance of the model to support the development of MSI status detection technology for colon cancer.

## 5 Conclusion

We have developed a two-stage DL method for diagnosing the MSI status of colon cancer based on CT, which involves segmentation followed by diagnosis. We have shown its effectiveness through experiments. However, further training with more data is required to verify its diagnostic skills in actual clinical settings.

## Data Availability

The raw data supporting the conclusions of this article will be made available by the authors, without undue reservation.
