# Correction: A two-stage deep learning prediction system for colon cancer microsatellite instability status using CT images

**DOI:** 10.3389/fonc.2026.1844233

**Published:** 2026-04-14

**Authors:** Songlin Cui, Xin Xiong, Xudong Yang, Jianfeng He, Tao Shen

**Affiliations:** 1 Faculty of Information Engineering and Automation, Kunming University of Science and Technology, Kunming, China; 2 Department of Colorectal Surgery, The Third Affiliated Hospital of Kunming Medical University, Kunming, China

**Keywords:** microsatellite instability, colon cancer, deep learning, segment anything, LORA, contrastive learning

An incorrect number was provided for The Yunnan Province Young and Middle aged Academic and Technical Leaders Project. The correct number is “202405AC350003”.

The original version of this article has been updated.

